# A continuum multi-species bacterial growth model with a novel interaction scheme

**DOI:** 10.1007/s00419-026-03160-y

**Published:** 2026-07-22

**Authors:** Felix Klempt, Hendrik Geisler, Meisam Soleimani, Philipp Junker

**Affiliations:** Institute of Continuum Mechanics, Leibniz University Hannover, Hannover, Germany

**Keywords:** Bacterial populations, Multi-species, Bacterial interaction, Continuum model

## Abstract

Biofilms are complex structures which are inhabited by numerous amount of different species of microorganisms. Due to their ubiquity, they influence human life on an everyday basis. It is therefore important to understand the interactions between different bacterial populations within a biofilm and their reactions to outside conditions. For this purpose, mathematical models and *in silico* experiments have proven themselves to be fundamental. In combination with *in vitro* and *in vivo* experiments, they can give more insights and focus researchers’ attention, reducing costs in the process. In this work, a comprehensive multi-species continuum-based model for the development of bacterial populations is presented. This model is capable of replicating a variety of different bacteria interactions with an arbitrary number of species, while still being comprehensive to encourage usage by researchers less familiar with mathematical modeling. In addition to a nutrient source, antibiotic agents and their effect on the biofilm can also be depicted. The model is derived using Hamilton’s principle of stationary action, ensuring thermodynamic consistency automatically. The numerical examples demonstrate the model’s capability to qualitatively reproduce complex interaction patterns.

## Introduction

The necessity to model and simulate the interaction between different bacterial species stems from the ubiquity of microbial biofilms. It is estimated that half of the existing biomass are prokaryotes [6, 30]. Much of that mass is located in dense biofilm communities, containing hundreds of species, i.e., upwards of 500 different species in dental biofilms [10, 18]. In these multi-species systems, complex interactions and relationships between different species emerge [8, 23]. The first, theoretical interaction is Neutralism, if biofilms do not interact at all with each other. With a lack of enough resources, i.e., space, nutrients or other necessary compounds, different species of bacteria have to compete with each other. Often times, the bacterial communities are then dominated by one species, but other ones persist [8]. If both species benefit from the presence of one another, it is called Protocooperation. This has often been demonstrated [8]. Other interactions are Allelopathy [25], where one species produces chemicals, which in turn influence the others. This can lead to Ammensalism, where one species harms the other and Commensalism, where one species benefits from the other. These interactions lead to the stabilization of the biofilm [8]. To control harmful and use beneficial bacterial species, an understanding of the underlying principles is necessary, but the large number of interactions pose a challenge for *in vitro* experiments [24]. In addition to species interacting with each other, biofilms are characterized by their spatial structure as well as detachment and attachment processes at the biofilm surface. Spatial structures in biofilms are induced by the diffusion of suspended substances such as nutrients, antibiotics, signal molecules or oxygen. This gradient of substances induces then the growth of different species at different places in the biofilm, resulting in an even more heterogenetic structure. While both of the effects are invaluable for a comprehensive description of biofilm, this work is focused on the description of the interaction of different bacteria species on a single material point, not on the overall structure which is evolving macroscopically. On its own it is not capable of depicting gradient based behavior. For this, it can be combined with non-local models such as [11] to display complex behavior in addition to the complex interactions inside of the biofilm. The model presented has the following key novelties which makes it a suitable extension to existing, non-local biofilm growth models. These novelties are:

- due to the derivation of the model through Hamilton’s principle of stationary action, the model is inherently thermodynamically consistent. It is based on the foundations of physics.
- straightforward parameter identification. Key parameters have a direct biological interpretation, which simplifies calibration. For instance, the viscosity η represents the metabolic inertia (or reaction delay) of a species, allowing the model to distinguish between fast-responding and slow-adapting populations based on simple growth curves.
- the kinetics of bacterial growth is modeled. Fast and slow growing species can be investigated in the same simulation.
- even though the model is simple in its mathematical formulation, complex behavior can be described.

Mathematical models can provide an environment where hypotheses can be tested rapidly and connections between different aspects can be drawn. Additionally, an *in silico* experiment, i.e., a simulation, takes considerably less time than an *in vitro* or an *in vivo* experiment. Models based on mathematical descriptions have thus proven themselves to be a viable tool in analyzing complicated processes [3]. Prominent modeling approaches are Individual Based Modeling (IbM), cellular automaton (CA) and continuum-based modeling. IbM seems to be the natural approach since bacterial populations themselves consists of individual cells, which can be seen as particles. Behaviors like cell divisions and interactions between two cells can be modeled directly as an interaction between two individual particles. There exist several open-source software systems using this approach, such as BacSim [13], Infobiotics Workbench [2], iDynoMiCS [15], Simbiotics [21], and NUFEB [16]. IbM models containing multiple species have been used to study the effects of EPS [12] as well as detachment and sloughing [31, 32] on multi species biofilm. In combination with the continuum approach, physical factors, like nutrient gradients, have been included [20, 23]. To avoid the stochastic effects resulting from the randomized placement of the individual particles in the modeling approach of IbM, the same simulation has to be repeated or it can be combined with cellular automata rules, see [14]. Multi-species models based fully on cellular automata are, for example, [19, 27, 28]. Another way of modeling biofilm is the continuum approach. Here, the incorporation of governing laws from physics comes naturally. Thermodynamic consistency can be assured automatically when using the right modeling approach. The thermodynamics and mechanics of growth are discussed in detail by [4] and [17]. Additionally, continuum-based models are deterministic, stochastic effects are thus not present and models do not need additional runs to average out random effects before conclusions can be drawn. Although the derivation of these models can be more complex, analytical studies can be performed to compare and characterize model results [1]. The complexity of these models makes them very predictive, but also hard to understand, especially for researchers not familiar with material modeling. Easier models can focus the attention on more important and relevant aspects of biofilm formation and growth, as well as species interaction. Multi-species continuum-based models in literature, which are suitable to model an arbitrary number of species, i.e., [1, 29], link the species via shared resources as space and nutrient available. This means that only competition between the species can be depicted. Models, which can depict Protocooperation, solve the problem of interaction with an additional interaction variable, which developes where one or both species are present and enhance the growth process of the other species [5, 26]. For a small number of species, i.e., dual-species systems, this is a suitable approach, but since for every interaction between two species an interaction variable is necessary, the number of internal variables and consequently the computational cost becomes unreasonable when the number of species increases.

In this work, a continuum model is presented which provides a framework for integrating a theoretically arbitrary number of species into the bacterial population. It is capable of displaying a wide variety of possible interactions from mutually inhibitory species to a symbiotic relationship. The integration of interaction is done via a constant interaction matrix. Thus, no additional internal variables need to be introduced. The model is derived from Hamilton’s principle of stationary action, ensuring thermodynamic consistency. The energy density function is modeled to be comprehensive. The complexity of the model stems from the dissipation function, which is formulated as a function of the product of the internal variables. It thus interconnects the governing evolution equations leading to complex material behavior.

The article is structured as follows. In Sect. 2, the mathematical derivation of the model is presented. The necessary variables and assumptions are introduced and the governing equations are shown. In Sect. 3, a numerical study displaying the behavior of the models with different numbers of species, initial conditions and parameters is presented to show the versatility of the model. The last chapter, Sect. 4, gives a short conclusion and an outlook for future investigations possible with this model.

## Mathematical description

In this work, a comprehensive model for bacterial growth, death and interaction is presented. An arbitrary number of species with interactions can be modeled. The model is derived based on Hamilton’s principle of stationary action. Hamilton’s principle provides a physically sound way of material modeling, in particular, the automatic fulfillment of the first and second law of thermodynamics through the derivation of the model from a potential [9]. Thus, the bacterial growth model presented in this work is thermodynamically consistent. In addition, necessary constraints can be considered in Hamilton’s principle simply by adding corresponding potentials. Hamilton’s principle for a quasi-static and isothermal process is given by [9, 11] as

1 $$\begin{aligned} \mathcal {H} = \int \limits _{\tau }(\mathcal {G} + \mathcal {C} + \mathcal {D})\; \textrm{dt} \rightarrow \underset{\boldsymbol{\xi }, \gamma }{\textrm{stat}}. \end{aligned}$$

The Hamilton functional $\mathcal {H}$ consists of three parts: the total potential $\mathcal {G}$, the constraint functional $\mathcal {C}$ and the dissipation energy $\mathcal {D}$. Hamilton’s principle states that $\mathcal {H}$ is stationary for all thermodynamic state variables. In this case, the thermodynamic state variables are the internal variables $\boldsymbol{\xi }$ and the Lagrange multiplier γ.

In the following, the individual parts are described in more detail. The total potential $\mathcal {G}$ is given as2with the Helmholtz free energy Ψ, the density $\rho _0$, the body force $\boldsymbol{f}^{\star }$, the traction force $\boldsymbol{t}^{\star }$ and the displacements $\boldsymbol{u}$. No external forces are applied to this continuum model. Therefore, the external forces $\boldsymbol{f}^{\star }$ and $\boldsymbol{t}^{\star }$ are set to zero.

The constraint functional

3 $$\begin{aligned} \mathcal {C} {:}{=}\int \limits _{\Omega _0} \, \gamma \, (f(\boldsymbol{\xi })) \; \textrm{dV}, \end{aligned}$$

allows incorporation of the holonomic constraint $f(\boldsymbol{\xi })$ with the Lagrange parameter γ.

The dissipated energy is given as

4 $$\begin{aligned} \mathcal {D} {:}{=}\int \limits _{\Omega _0} \textrm{D}_{\textrm{diss}} \; \textrm{dV}. \end{aligned}$$

with the volume-specific dissipated potential $\text {D}_\text {diss}$.

To arrive at a sensible description of a predictive model for bacterial growth and death, suitable model assumptions have to be made, which will be explained in the following.

The set of internal variables $\boldsymbol{\xi }$ is given as

5 $$\begin{aligned} \boldsymbol{\xi }= \begin{pmatrix} \boldsymbol{\phi }\\ \boldsymbol{\psi }\end{pmatrix}. \end{aligned}$$

The volume fraction $\phi _i \; i \in \{1, 2, \cdots , n \}$, which indicates the volume covered by each of *n* species of bacteria, as well as the empty space $\phi _0$, is listed in the vector

6 $$\begin{aligned} \boldsymbol{\phi }= \begin{pmatrix} \phi _0\\ \phi _1\\ \phi _2\\ \vdots \\ \phi _n \end{pmatrix} \; \textrm{with} \; \phi _i \in [0,1], \; \forall i. \end{aligned}$$

In order to model the growth as well as the death of bacteria, a second state variable per species is necessary. The percentage of living bacteria of the *n*th species is given by

7 $$\begin{aligned} \boldsymbol{\psi }= \begin{pmatrix} \psi _1\\ \psi _2\\ \vdots \\ \psi _n \end{pmatrix}\; \textrm{with} \; \psi _i \in [0,1], \; \forall i. \end{aligned}$$

Therefore, the volume covered by living bacteria of species *i* is

8 $$\begin{aligned} \bar{\phi }_i {:}{=}\phi _i \, \psi _i \; \textrm{with} \; \forall i. \end{aligned}$$

The amount of dead cells is consequently $\tau _i {:}{=}\phi _i \, (1-\psi _i) \; \textrm{with} \; i \in \{1, 2, \cdots , n \}$. Since the total volume of all bacteria plus the empty space cannot exceed the available volume, the holonomic constraint

9 $$\begin{aligned} f = \sum _{l=0}^n \phi _l - 1 = 0 \end{aligned}$$

has to be fulfilled at all times.Since the model is formulated as a material point model, interpreting the empty space as physical voids or channels within the barterial population would be inappropriate. The empty space variable $\phi _0$ rather serves as a numerical auxiliary quantity used to enforce the holonomic volume constraint given by Eq. (9) The nutrients are modeled by a given variable $\textrm{c}^{\star }$, and the antibiotics by the given variable $\alpha ^{\star }$. Both are possibly time-dependent. Since living bacteria thrive with nutrients and the percentage of living bacteria reduces with antibiotics present, the energy density function is modeled as

10 $$\begin{aligned} \Psi = - \frac{1}{2} \textrm{c}^{\star } \; \bar{\boldsymbol{\phi }} \cdot \boldsymbol{A}\cdot \bar{\boldsymbol{\phi }} + \frac{1}{2} \alpha ^{\star } \; \boldsymbol{\psi }\cdot \boldsymbol{B}\cdot \boldsymbol{\psi }. \end{aligned}$$

The quadratic approach is chosen to allow for a linear dependency between nutrients and $\bar{\phi }_i$ and antibiotics and $\psi _i$. Additionally, the quadratic approach ensures the convexity of the free energy density. The symmetric growth coefficient matrix

11 $$\begin{aligned} \boldsymbol{A}= \begin{pmatrix} a_{11} &  a_{12} &  \cdots &  a_{1n}\\ a_{12} &  a_{22} &  \cdots &  a_{2n}\\ \vdots &  \vdots &  \ddots &  \vdots \\ a_{1n} &  a_{2n} &  \cdots &  a_{nn} \end{pmatrix} \end{aligned}$$

and the antibiotic sensitivity matrix

12 $$\begin{aligned} \boldsymbol{B}= \begin{pmatrix} b_{1} &  0 &  \cdots &  0\\ 0 &  b_{2} &  \cdots &  0\\ \vdots &  \vdots &  \ddots &  \vdots \\ 0 &  0 &  \cdots &  b_{n} \end{pmatrix}. \end{aligned}$$

allow to specify the effect of nutrients and antibiotics on each species. Furthermore, the off-diagonal entries of the matrix $\boldsymbol{A}$ describe how the different species of bacteria interact with one another, i.e., if they promote or impair each others growth. While in principle the entries of the matrices can have any form, i.e., be a function of any parameters or variables of the model, for simplicity reasons a zero-order Taylor series, i.e., a constant, is chosen to represent the interactions with nutrients and antibiotics. Hereby, a positive value indicates an equally favorable, a negative value an equally disadvantageous interaction. The interaction between two species is consequently modeled with a single scalar with no need for additional state variables as in [26]. This modeling choice vastly decreases simulation time.

The dissipated energy is modeled as

13 $$\begin{aligned} \mathrm {D_{diss}} {:}{=}\frac{\partial \Delta ^s}{\partial \dot{\boldsymbol{\xi }}} \cdot \boldsymbol{\xi }. \end{aligned}$$

For more information on modeling assumptions concerning energy dissipation in the Hamiltonian framework, see [9]. The dissipation function is modeled as

14 $$\begin{aligned} \Delta ^s = \Delta ^s(\dot{\bar{\boldsymbol{\phi }}}, \dot{\boldsymbol{\phi }}) = \frac{1}{2} \; \dot{\bar{\boldsymbol{\phi }}} \cdot \boldsymbol{\eta }\cdot \dot{\bar{\boldsymbol{\phi }}} + \frac{1}{2} \; \dot{\boldsymbol{\phi }} \cdot \boldsymbol{\eta }\cdot \dot{\boldsymbol{\phi }}. \end{aligned}$$

with the diagonal viscosity matrix

15 $$\begin{aligned} \boldsymbol{\eta }= \begin{pmatrix} \eta _1 &  0 &  \cdots &  0\\ 0 &  \eta _2 &  \cdots &  0\\ \vdots &  \vdots &  \ddots &  \vdots \\ 0 &  0 &  \cdots &  \eta _n \end{pmatrix}. \end{aligned}$$

In this context, the viscosity parameter η does not describe a fluid mechanical property of the biomass, but rather acts as a kinetic resistance governing the rate of evolution. Biologically, $\eta _i$ represents the metabolic inertia of the *i*-th species. A high value corresponds to a species that reacts slowly to environmental changes (e.g., due to complex metabolic switching), whereas a low value describes a rapidly adapting population. This ensures that the population growth follows a physical timescale rather than occurring instantaneously. The dissipation function is the sum of two parts. The first part describes the dissipation due to the change in living bacteria species. Due to the living species being the product of the two state variables $\bar{\phi _i} = \phi _i \psi _i$, the rates of the internal variables $\dot{\phi }$ and $\dot{\psi }$ are directly linked together. This allows for complicated, non-linear effects in the model. Using only the first term of the dissipation function would lead to a rank-deficient system of differential equations. The second term depending only on the rate of the volume fraction of each species leads to a full rank. The overall resulting behavior is rate-dependent.

By evaluating Hamilton’s principle, the strong form of the evolution equations are found as

16 $$\begin{aligned} \delta _{\phi } \mathcal {H} = 0 \; \forall \delta \phi \; \Leftrightarrow \; 0&= - c^{\star } \psi _i \left( a_{ii} \bar{\phi }_i+ \sum _j^{n-1} a_{ij} \bar{\phi }_j \right) + \eta _i (\dot{\phi _i}\psi _i^2 +\bar{\phi }_i \dot{\psi }_i + \dot{\phi }_i) + \gamma \end{aligned}$$

17 $$\begin{aligned} \delta _{\psi } \mathcal {H} = 0 \; \forall \delta \psi \; \Leftrightarrow \; 0&= -c^{\star } \phi _i \left( a_{ii} \bar{\phi }_i+ \sum _j^{n-1} a_{ij} \bar{\phi }_j \right) + \alpha ^{\star } \psi _i b_i + \eta _i (\dot{\psi _i}\phi _i^2 + \bar{\phi }_i \dot{\phi }_i) + \gamma \end{aligned}$$

18 $$\begin{aligned} \delta _{\gamma } \mathcal {H} = 0 \; \forall \delta \gamma \; \Leftrightarrow \; 0&= \sum _{l=0}^n \phi _l - 1 \end{aligned}$$

The variable describing the empty space $\phi _0$ is described through the constraint in Eq. (9). The model was implemented using Wolfram Mathematica [7] in an implicit framework on a material point. Therefore, the governing Eqs. (16), (17) and (18) are solved using Newton’s method at each time step. The volume constraint Eq. (9) is enforced by means of a Lagrange multiplicator γ. To enforce $\phi _i, \psi _i \in [0,1]$, the barrier method is used with a penalty parameter $K_{p,i} =\eta _i \; 10^{-4}$ [22].

## Numerical results

Several numerical experiments were performed to investigate the behavior of the model. First, a model with two species was chosen. This minimal model helps in understanding the interaction between different variables. For further investigation, a four species model was implemented. The four species model allows for a more thorough investigation of the model as well as showing more complex behaviors. The time step size Δt is set constant to $\Delta t = 10^{-4} \textrm{Time}$ throughout the numerical experiments. The unit of time can be chosen to represent an appropriate timescale for bacterial growth and has to be determined in subsequent *in vitro* experiments.

### Two species

For the two species model, five cases are investigated. They differ in the parameters listed in Table 1. It should be noted that the parameters chosen in this numerical study are representative, normalized values intended to investigate the qualitative behavior of the model stability and interaction patterns, rather than fitting a specific biological organism.

**Table 1:** Values for the parameters of the simulations performed with two species present. The variables describing the nutrients $\textrm{c}^{\star }$ and the antibiotics $\alpha ^{\star }$ are left constant at $\textrm{c}^{\star } = {100}\,\frac{\textrm{J}}{\textrm{m}^{3}}$ and $\alpha ^{\star } = {10}\,\frac{\textrm{J}}{\textrm{m}^{3}}$

Variable	Unit	Case 1	Case 2	Case 3	Case 4	Case 5	Case 6
${a}_{11}$	[-]	2	1	1	1	1	1
${a}_{12}$	[-]	0	0	1	0	0	-1
${a}_{22}$	[-]	1	1	1	1	1	1
${b}_{1}$	[-]	0	0	0	1	1	0
${b}_{2}$	[-]	0	0	0	2	2	0
${\eta }_{1}$	$[\frac{\textrm{kg}}{\textrm{m}\, Time}]$	1	1	1	1	1	1
${\eta }_{2}$	$[\frac{\textrm{kg}}{\textrm{m}\, Time}]$	1	2	2	2	2	2
initial $\phi _{1}$	[-]	0.2	0.2	0.2	0.2	0.25	0.2
initial $\phi _{2}$	[-]	0.2	0.2	0.2	0.3	0.3	0.2

**Fig. 1:**
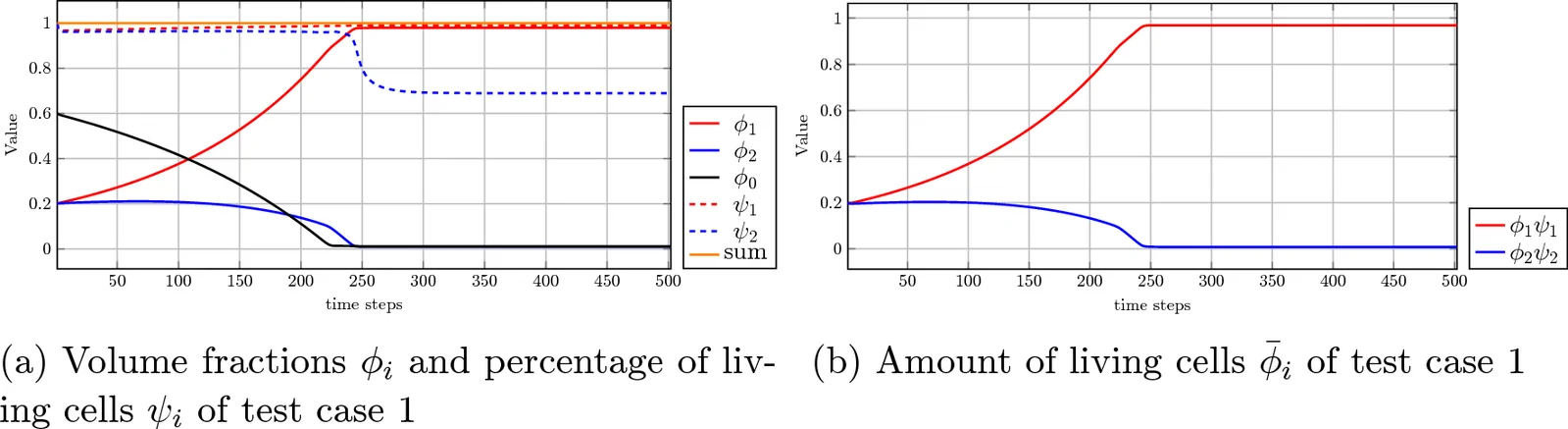
Results of the test case with two species with different growth parameters. The species only interact through the space available and don’t enhance each other’s growth. Both species are fully resistant to antibiotics

**Fig. 2:**
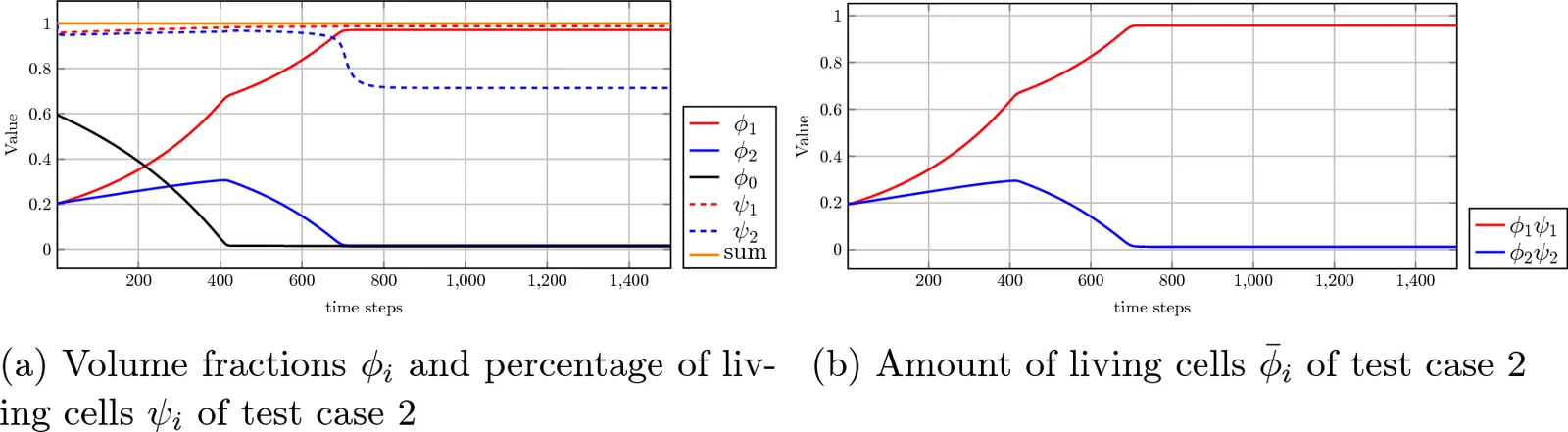
Results of the test case with two species with different viscosities. The species only interact through the space available and do not enhance each other’s growth. Both species are fully resistant to antibiotics

The results from the first test case are depicted in Fig. 1a and b. Figure 1a shows the plot of the state variables $\boldsymbol{\phi }$ and $\boldsymbol{\psi }$ over time. The volume of each species $\phi _i$ is given with a solid colored line. The empty space $\phi _0$ is shown in black. The dashed line represents the percentage of living bacteria of a given species $\psi _i$. In this first test case, the only interaction between both species is due to the limited amount of space available, since the off-diagonal terms of the growth parameter matrix $\boldsymbol{A}$ are set to zero. The diagonal entries of the growth parameters matrix are chosen to be $a_{11} > a_{22}$. This results in a faster growth rate of species 1. Once the empty space converges to zero, kinks in the graphs emerge and the volume fraction $\phi _2$ drops down rapidly. As soon as the volume fraction of species 2 reaches a value close to zero, its percentage of living cells $\psi _2$ drops down. Similar effects can be observed in the results of test case 2 in Fig. 2a and b. In this test case, the viscosity of the species $\eta _i$ differs. A higher viscosity leads to a slower reaction of the bacterial species. The simulation time is thus elongated to be 1500 time steps. The aforementioned model behaviors from case 1 are also visible and even more prominent. While in case 1 the amount of species 2 $\phi _2$ is already at a low percentage of around 10% and still declining before the empty space reaches zero. In case 2, $\phi _2$ is at roughly 30% and ascending. The different amounts of species 2 present, result in different reaction from species 1. In the first case, the slope of the $\phi _1$-curve changes only slightly, while in case 2 a prominent kink is observable. The drop in the percentage of living bacteria of species 2 $\psi _2$ is comparable for both cases, as is the converged steady state solution.

**Fig. 3:**
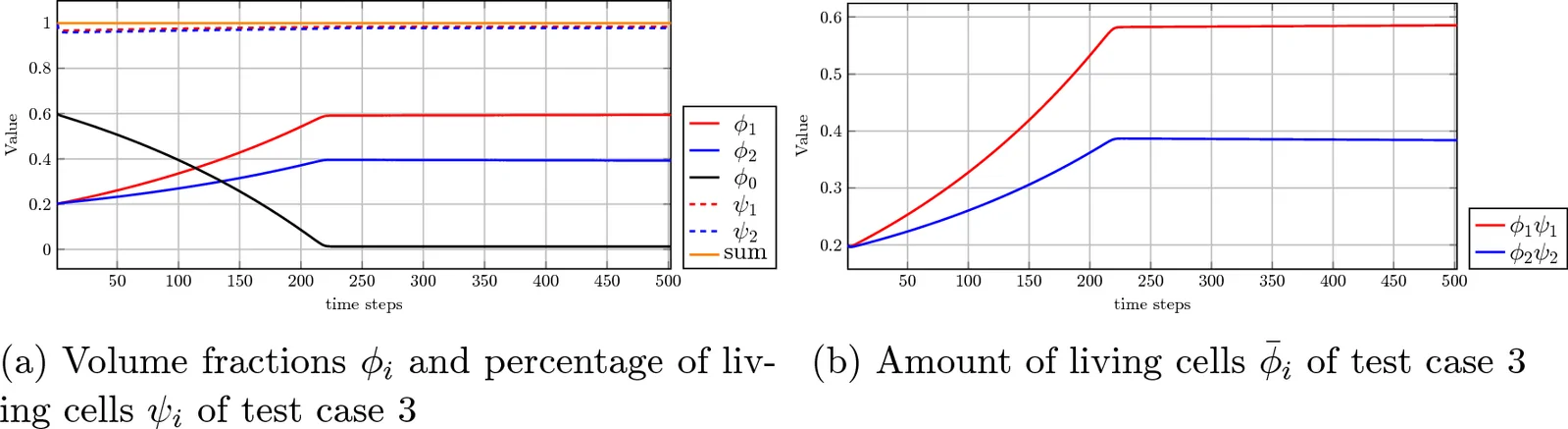
Results of the test case with two species. The species interact and enhance each others growth. Both species are fully resistant to antibiotics

When interactions between the species are enabled via $a_{ij} \ne 0 \; \textrm{for} \; i \ne j$, as it is in test case 3, the survival of the other species is favorable for both species. An equilibrium between the two species emerges, where none gets extinguished. This can be observed in the result of test case 3 in Fig. 3a and b.

**Fig. 4:**
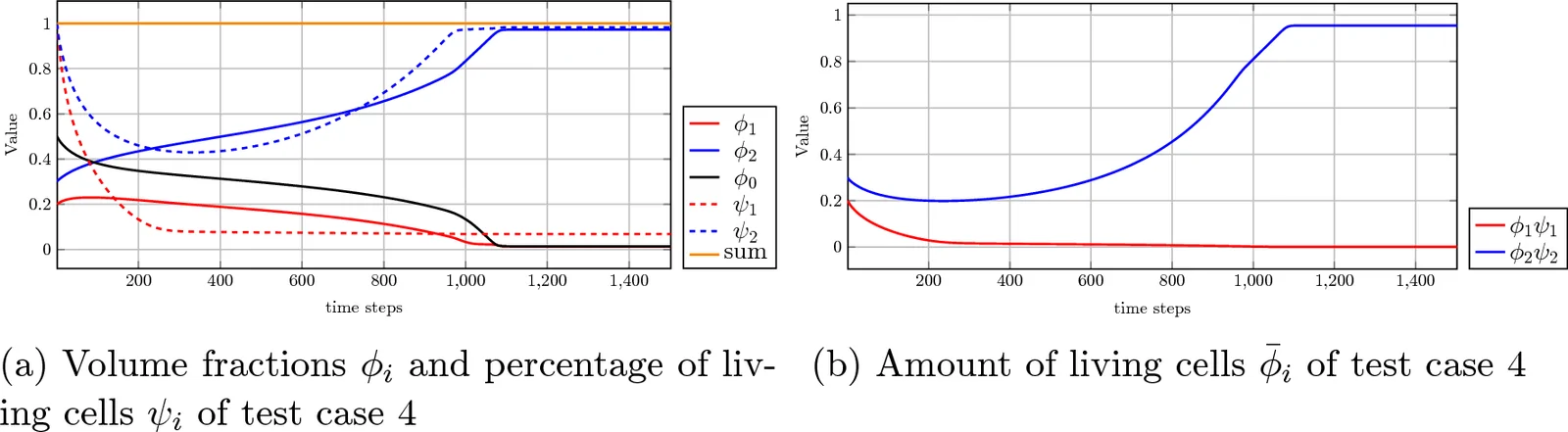
Results of the test cases with sensitivity of antibiotics. The initial volume fractions of species $\phi _i$ differ. Species 1 starts with an initial volume fraction of $\phi _1=0.2$, species 2 with $\phi _2=0.3$

**Fig. 5:**
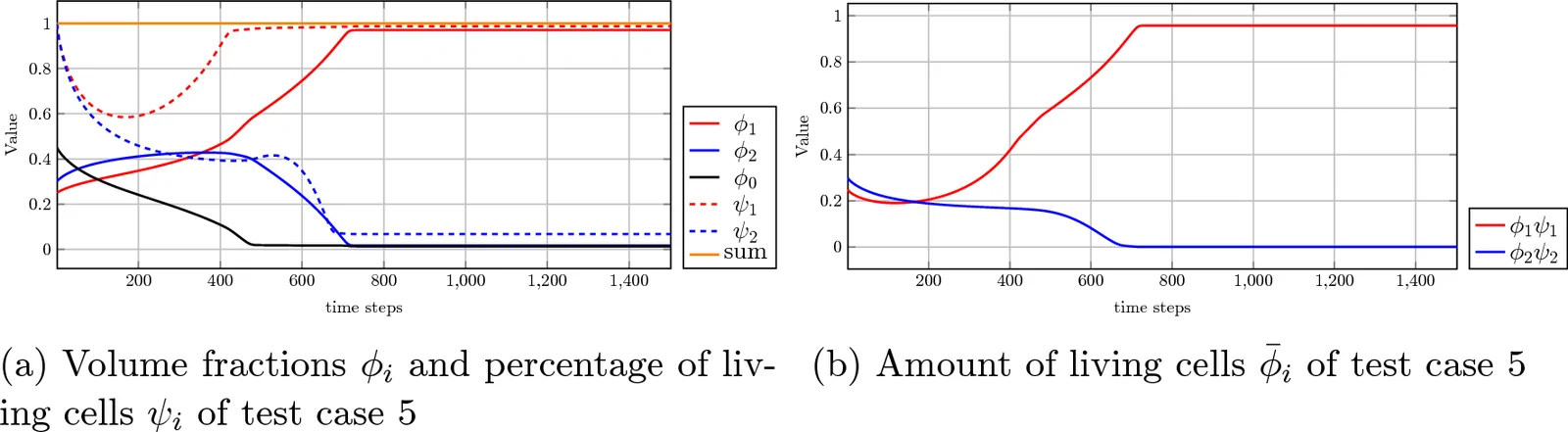
Results of the test cases with sensitivity of antibiotics. The initial volume fractions of species $\phi _i$ differ. Species 1 starts with an initial volume fraction of $\phi _1=0.2$, species 2 with $\phi _2=0.3$

**Fig. 6:**
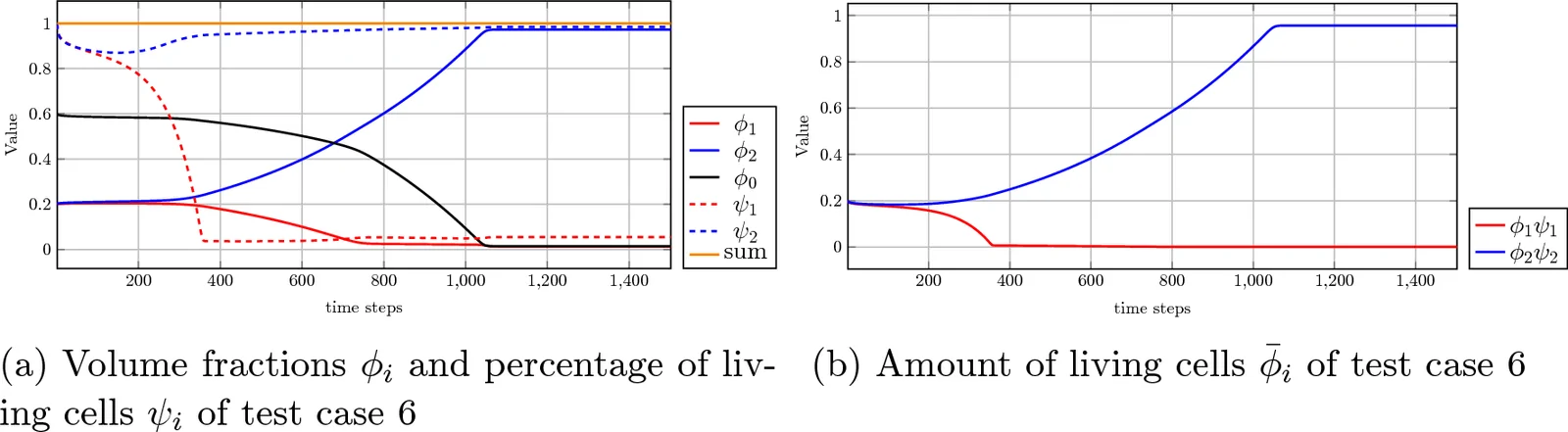
Results of the test cases with negative entry in the growth coefficient matrix

For antibiotics sensitive bacterial species, longer simulation times are necessary as well. It can be observed in Figs. 4a and 5a, that the percentage of living species $\psi _i$ drops initially. Although the sensitivity for antibiotics of species 2 is higher than that of species 1, due to the higher initial condition, species 2 can prevail over species 1 in the fourth test case. If the difference of the initial amount of bacteria is closer, as it is in test case 5, it is not enough and species 1 prevails instead. This can be seen in Fig. 5a. The amount of living bacteria of each species $\bar{\phi _i} = \phi _i \psi _i$ can be observed for both cases in Figs. 4b and 5b. In all cases above, the interaction between different species is modeled with a positive integer to model cooperative behavior. To model competition, a negative value for the off-diagonal entries of the interaction matrix $\textbf{A}$ can be used. The results of this simulation are shown in Fig. 6. It can be observed, that the slower reacting species 2, i.e., the species with a higher viscosity $\eta _i$, comes out dominant. While a faster reaction is advantageous when reacting to nutrients, it can be detrimental when reacting negatively to another species. Early in the simulation, the percentage of living bacteria of species 1 $\psi _1$ drops of faster than the the living percentage of species 2 $\psi _2$. This leads to a difference in volume growth, where the species 2 with more living cells flourishes. The drop off in percentage of living cells is different from the experiment, where no interactions is present (c.f. Figure 1). There is no negative part in the driving force in these simulation, thus the percentage of living cells $\psi _i$ stays at a relatively high level.

Additional simulation with varying viscosities $\eta _i$ were performed. The results are listed in Appendix 1.

### Four species

In theory, an arbitrary number of species can be inserted into the model. For this study, a bacterial population consisting of four species is considered. The growth parameter matrix $\boldsymbol{A}$ stays the same throughout all numerical experiments in this chapter, as do the viscosities $\eta _i$, only the antibiotic sensitivity parameters $b_i$ as well as the nutrient concentration $c^{\star }$ and the concentration of antibiotics $\alpha ^{\star }$ are changed. The values for the symmetric growth coefficient matrix $\boldsymbol{A}$ and the diagonal viscosity matrix $\boldsymbol{\eta }$ are

19 $$\begin{aligned} \boldsymbol{A}= \frac{1}{2} \begin{pmatrix} 1 &  5 &  5 &  5\\ 5 &  1 &  3 &  3\\ {5} &  3 &  1 &  2 \\ 5 &  3 &  2 &  1 \end{pmatrix} \end{aligned}$$

and

20 $$\begin{aligned} \boldsymbol{\eta }= \begin{pmatrix} 0.8 &  0 &  0 &  0\\ 0 &  1.0 &  0 &  0\\ 0 &  0 &  1.5 &  0 \\ 0 &  0 &  0 &  2.0 \end{pmatrix} \frac{\textrm{kg}}{\textrm{m}\,\text {Time}}, \end{aligned}$$

respectively. The values for the remaining parameters of the four cases investigated are listed in Table 2.

**Table 2:** Values for the parameters of the simulations performed with four species present

Variable	Unit	Case 1	Case 2	Case 3	Case 4
${b}_{1}$	[–]	0.4	0.4	0.4	10
${b}_{2}$	[–]	0.3	0.3	0.3	2
${b}_{3}$	[–]	0.2	0.2	0.2	1
${b}_{4}$	[–]	0.1	0.1	0.1	0.01
initial $\phi _{1}$	[–]	0.02	0.02	0.02	0.02
initial $\phi _{2}$	[–]	0.02	0.02	0.02	0.02
initial $\phi _{3}$	[–]	0.02	0.02	0.02	0.02
initial $\phi _{4}$	[–]	0.02	0.2	0.02	0.02
nutrients $c^{\star }$	$[\frac{\textrm{J}}{\textrm{m}^{3}}]$	100	100	$50 + 50 \; \textrm{Sin}(500 t)$	100
antibiotics $\alpha ^{\star }$	$[\frac{\textrm{J}}{\textrm{m}^{3}}]$	10	10	10	$\textrm{Piecewise} \; \{0,\{100,t>500\}\}$

**Fig. 7:**
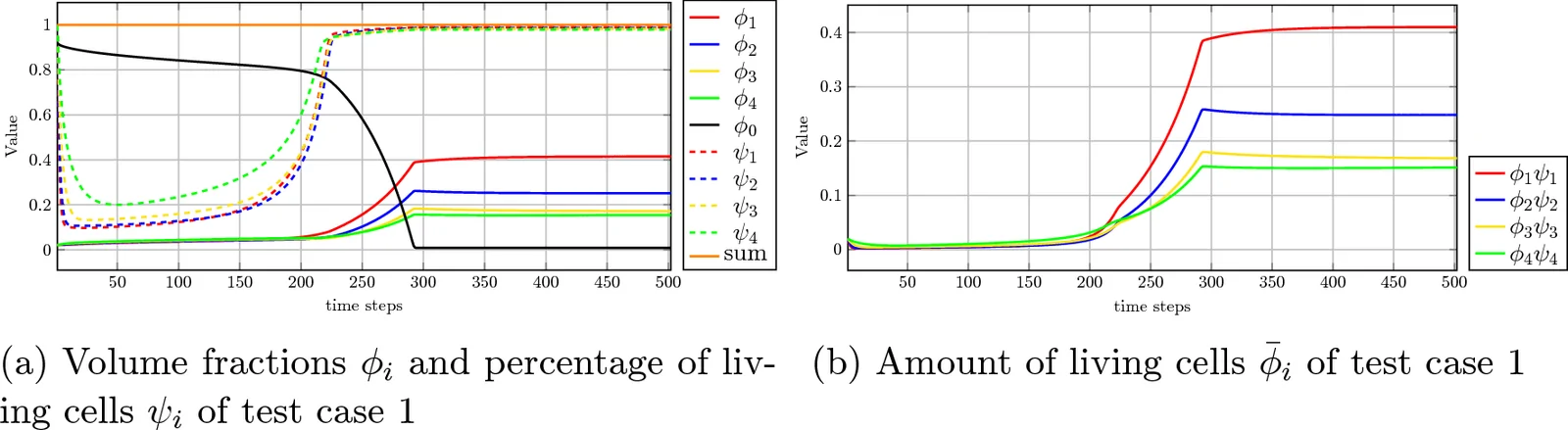
Result of the first test case with four species

**Fig. 8:**
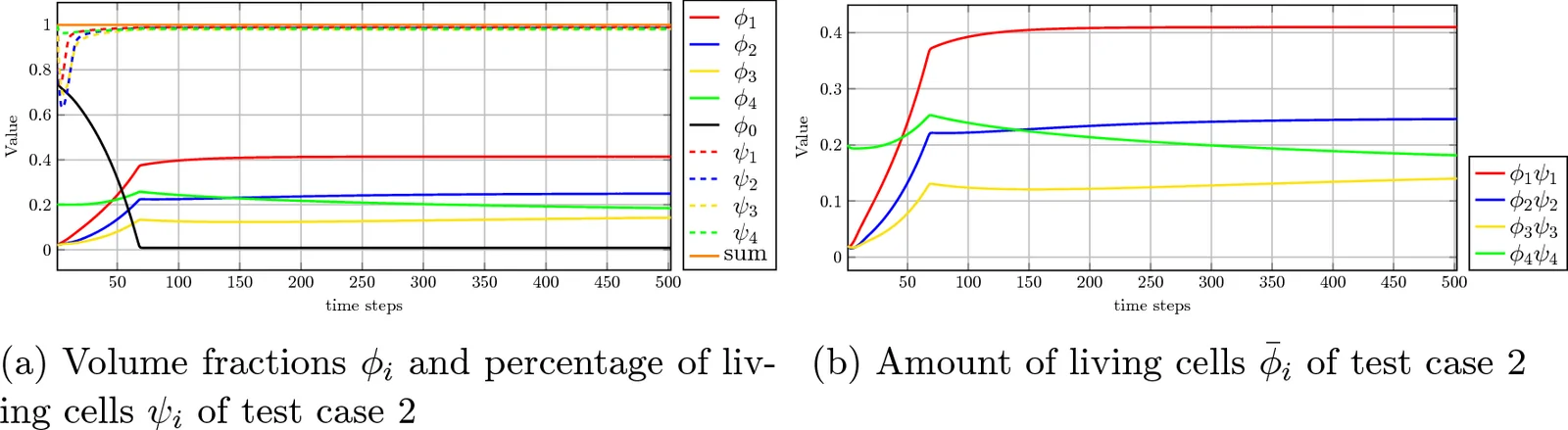
Result of the second test case with four species. The initial volume fractions of species $\phi _i$ differ. Species 1, 2 and 3 start with an initial volume fraction of $\phi _{1} = \phi _{2} = \phi _{3} = 0.02$, species 4 starts with $\phi _4 = 0.2$

**Fig. 9:**
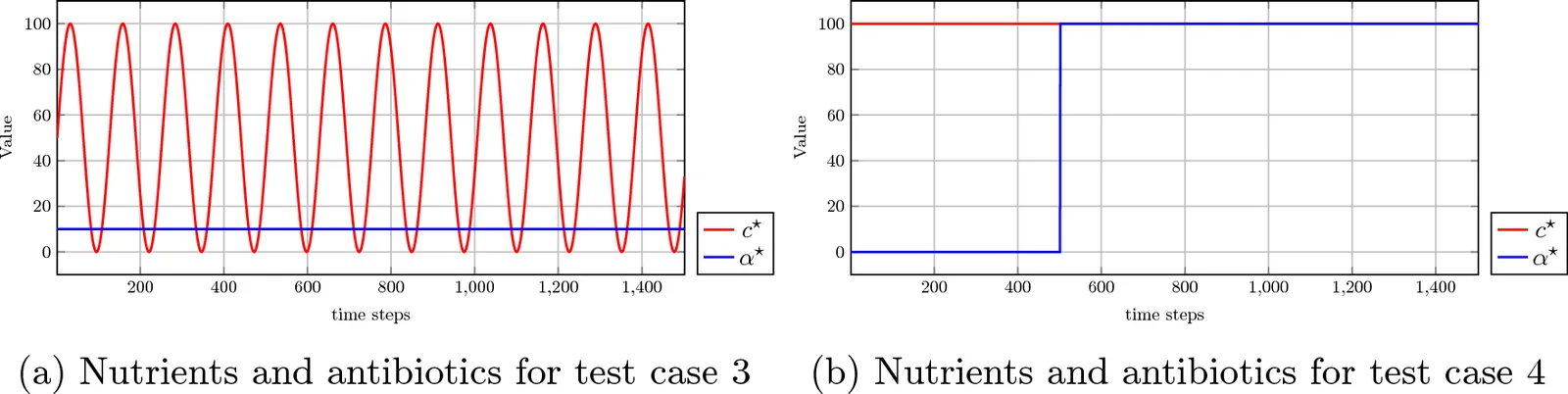
Functions with which nutrients and antibiotics are applied in test cases 3 and 4

**Fig. 10:**
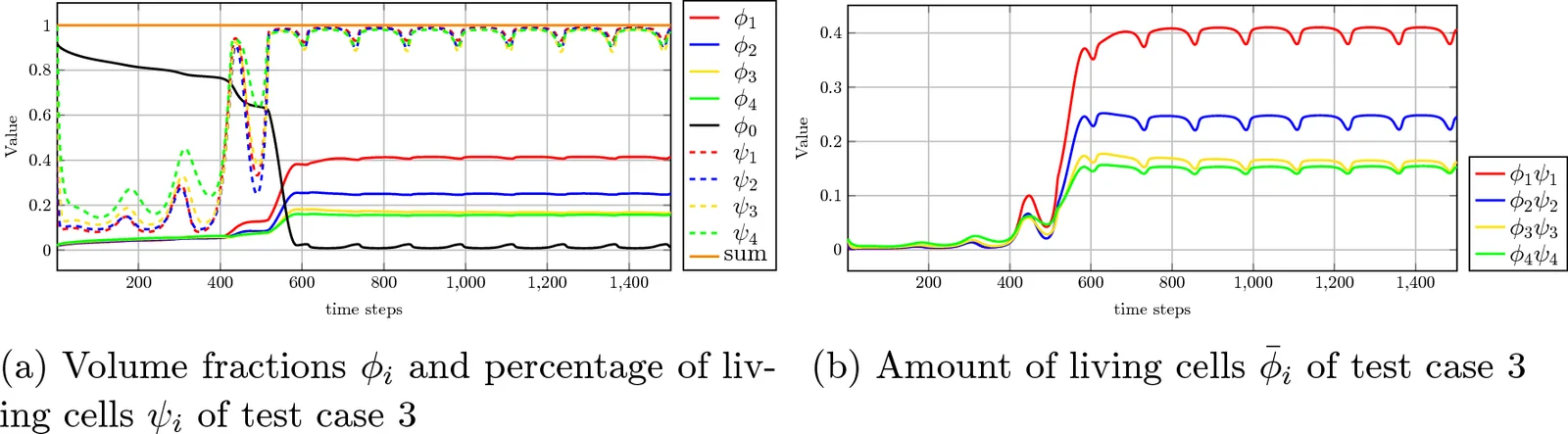
Result of the third test case with four species. Nutrients are applied with a sine function

In Figs. 7 and 8, the results can be seen. The first case shows all four species with the same initial amount of bacteria. Since the antibiotic sensitivity differs in all four species, with species 1 being the most sensitive and species 4 being the least sensitive, the initial drop in living percentage of each species $\psi _i$ is also different. Species 4 is the only species with a minimum of 20% of its cells still living. Due to the greater growing parameter of the first species, however, in the course of the simulation, the first species takes over as the dominant species. The different viscosities $\eta _i$ determine how fast each species grows. This can be seen in Figs. 7a and 8a, where the first species’ gradient in the beginning is much greater than that of each other species. The second case investigated here only differs in the initial conditions, i.e., the fourth species gets a head start in the simulation. Since there is now less free space available to all species, the equilibrium between all species is reached earlier. In addition to that, by the last time step, species 4 still has more volume than species 3, which, in the prior experiment, had more. For the nutrients, as well as the antibiotics, an arbitrary function can be implemented. In the following test case, the nutrients were applied with a Sine function (see Fig. 9a). The results can be seen in Fig. 10a and b. The general shape of the results roughly mimics these from Fig. 7a, with the exception of the fluctuation due to little (or no) nutrients in the system.

**Fig. 11:**
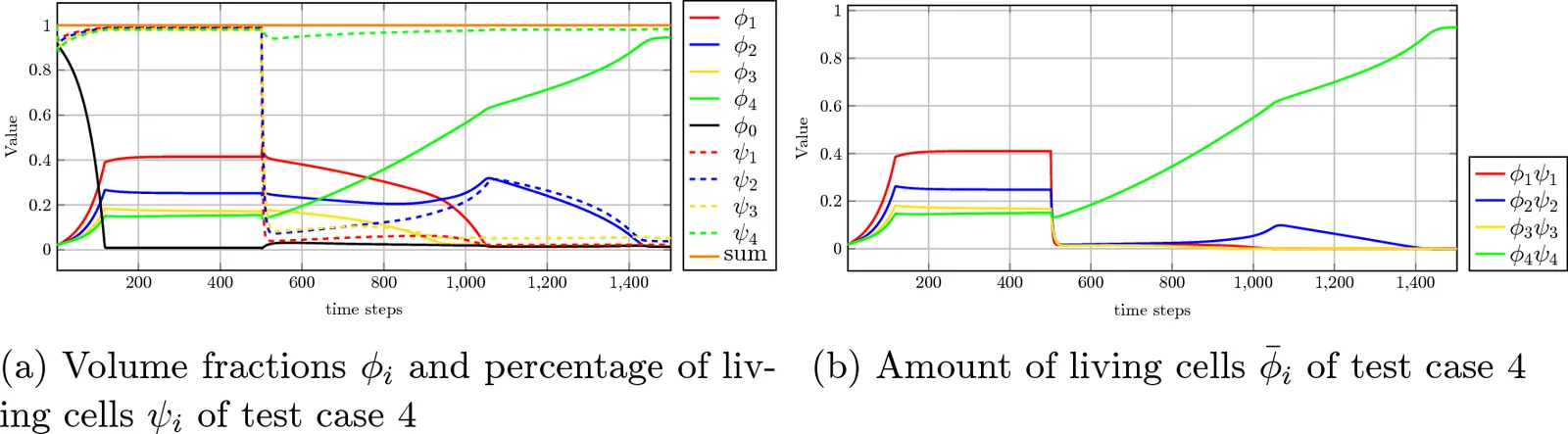
Result of the forth test case with four species. Antibiotics are applied with a step function at time step 500

For the final case investigated, the sensitivity towards antibiotics was adjusted. The first species now has a high sensitivity of $b_1 = 10$, while the fourth species is almost resistant against antibiotics with $b_4 = 0.01$. After one-third of the simulation time, antibiotics are inserted into the system (see Fig. 9b). Every species, except the resistant species 4, almost immediately dies, as can be observed in Fig. 11a and b. After the insertion of antibiotics, resistant species 4 takes over the space which was inhabited by the other species prior to the insertion. The second species, which is not as sensitive to antibiotics but has a smaller viscosity than species 4, i.e. $\eta _2 {<} \eta _4$, and is thus more reactive to changes in the system, regains some space but is ultimately dominated by the fourth species. In this example, cooperative behavior is not the dominating factor of species survival. The resistance against antibiotics is more important.

## Conclusion and outlook

In this work, a new multi-species model for growth in bacterial populations is presented. In principle, an arbitrary number of species can be described using this model. For the description, one internal variable is needed to describe the empty space as well as two additional internal variables for each species. In this work, bacteria species are modeled with the variables of the volume fraction of all, living and dead, bacterial cells ϕ, as well as the percentage of living cells inside the volume fraction ψ. This leads to the amount of living cells given by $\bar{\phi } = \phi \psi$. The interactions between each species are modeled with energies.

A dissipated function is introduced as a function of the living bacteria $\bar{\phi }$ and thus a product of the internal state variables, leading to a coupled system of evolution equations, capable of non-linear effects. The free energy density is modeled with little assumptions concerning the growth and death of bacteria. Nevertheless, the model is capable of producing complex results. Many behaviors as observed in multi-species bacterial interactions can be modeled. Additionally, the kinetics of bacterial growth is modeled. The reduced number of parameters of the model simplifies the pending validation of the model with *in vitro* experiments. The model is also suitable for further investigation of bacterial behavior, for example, accounting for stochastic properties. It can be used in combination with existing, non-local biofilm growth models to depict the complex interactions between different species of bacteria as well as structural properties of macroscopic biofilms (spatial heterogeneity) and the suspended substances within.

## Figures and Tables

**Fig. 12:**
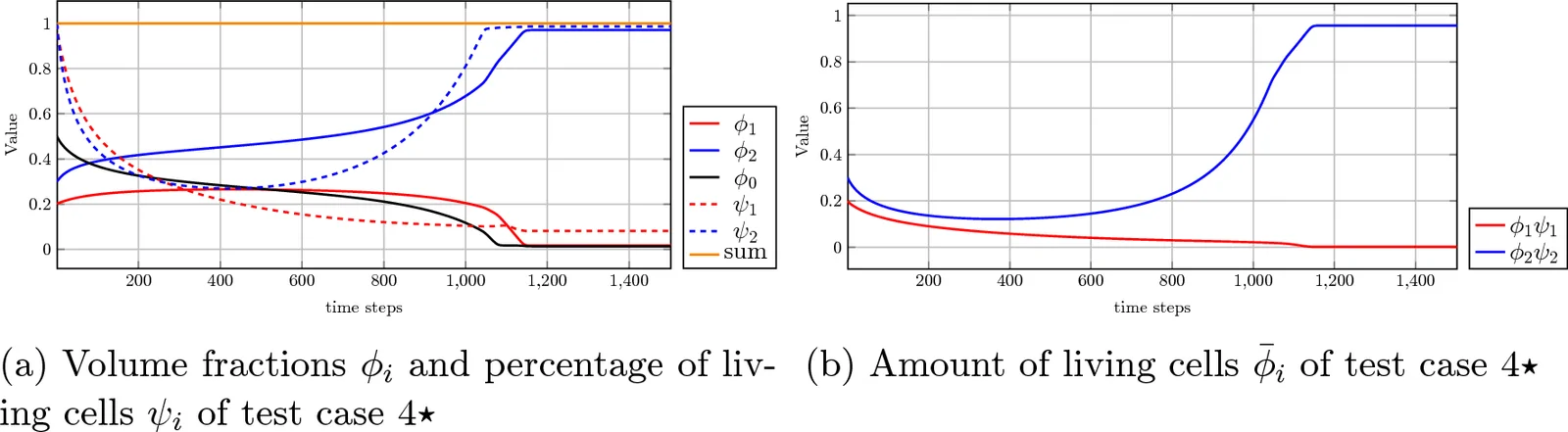
Results of the additional case with two species based on test case 4 with varying viscosites. The viscosities have been switched compared to case 4

**Fig. 13:**
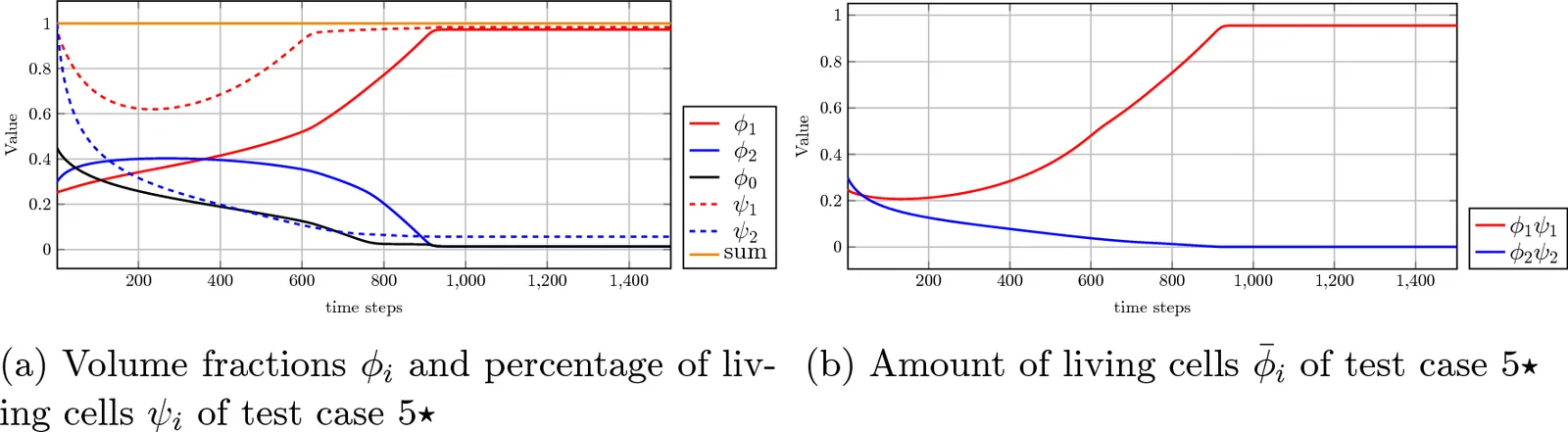
Results of the additional case with two species based on test case 5 with varying viscosites. The viscosities have been switched compared to case 5

**Fig. 14:**
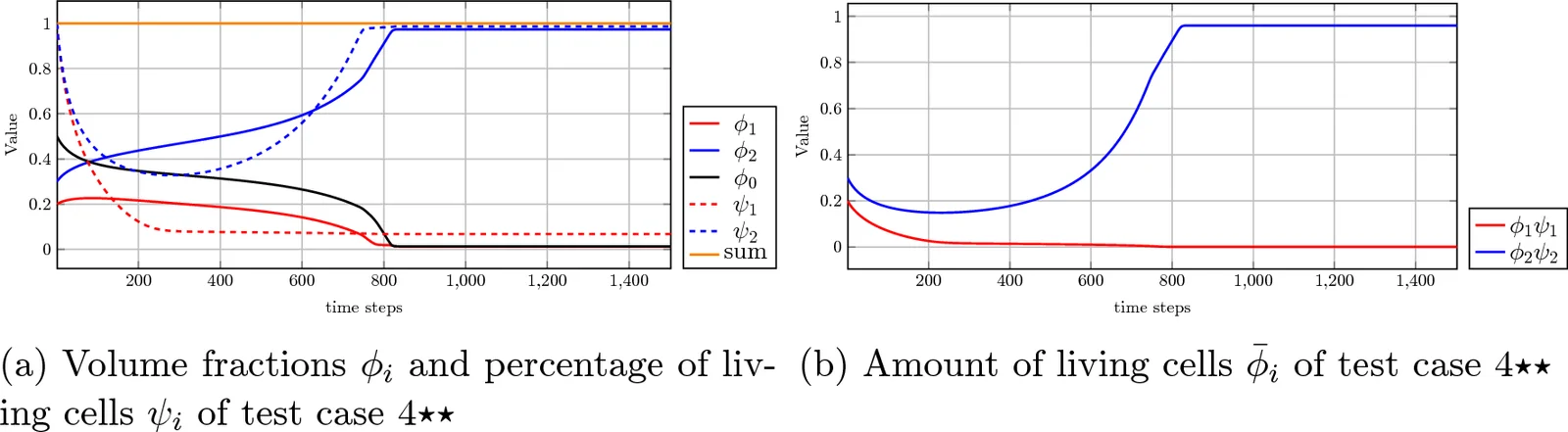
Results of the additional case with two species based on test case 4 with varying viscosities. The viscosities have been set equal

**Fig. 15:**
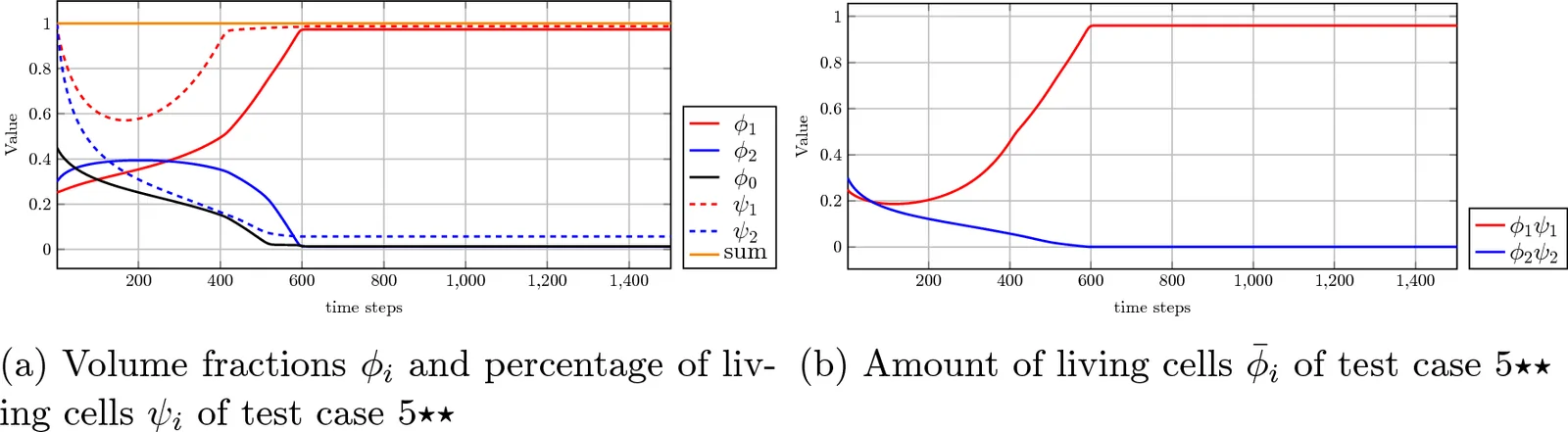
Results of the additional case with two species based on test case 5 with varying viscosites. The viscosities have been set equal

## Data Availability

No datasets were generated or analysed during the current study.

## Appendix 1

Additional simulation were performed to further investigate the behavior of the viscosities $\eta _i$. The simulations provided here are based on the cases 4 and 5 for the two species model respectively.

See Figs. 12, 13, 14, 15 and Table 3.

**Table 3:** Values for the parameters of the additional simulations performed with two species present. The variables describing the nutrients $\textrm{c}^{\star }$ and the antibiotics $\alpha ^{\star }$ are left constant at $\textrm{c}^{\star } = {100}\,\frac{\textrm{J}}{\textrm{m}^{3}}$ and $\alpha ^{\star } = {10}\,\frac{\textrm{J}}{\textrm{m}^{3}}$

Variable	Unit	Case 4${\star }$	Case 5${\star }$	Case 4${\star \star }$	Case 5${\star \star }$
${a}_{11}$	[–]	1	1	1	1
${a}_{12}$	[–]	0	0	0	0
${a}_{22}$	[–]	1	1	1	1
${b}_{1}$	[–]	1	1	1	1
${b}_{2}$	[–]	2	2	2	2
${\eta }_{1}$	$[\frac{\textrm{kg}}{\textrm{m}\,Time}]$	2	2	1	1
${\eta }_{2}$	$[\frac{\textrm{kg}}{\textrm{m}\,Time}]$	1	1	1	1
initial $\phi _{1}$	[–]	0.2	0.25	0.2	0.25
initial $\phi _{2}$	[–]	0.3	0.3	0.3	0.3
